# Verifying Safety Messages Using Relative-Time and Zone Priority in Vehicular Ad Hoc Networks

**DOI:** 10.3390/s18041195

**Published:** 2018-04-13

**Authors:** Sam Banani, Steven Gordon, Surapa Thiemjarus, Somsak Kittipiyakul

**Affiliations:** 1 School of Information, Computer, and Communication Technology, Sirindhorn International Institute of Technology, Thammasat University, Pathum Thani 12000, Thailand; sam@ict.siit.tu.ac.th (S.B.); somsak@siit.tu.ac.th (S.K.); 2 School of Engineering and Technology, CQUniversity, Cairns QLD 4870, Australia; 3 National Electronics and Computer Technology Center, Pathum Thani 12120, Thailand; surapa.thiemjarus@nectec.or.th

**Keywords:** vehicular ad hoc networks, wireless communications, prioritization, message, verification, basic safety message

## Abstract

In high-density road networks, with each vehicle broadcasting multiple messages per second, the arrival rate of safety messages can easily exceed the rate at which digital signatures can be verified. Since not all messages can be verified, algorithms for selecting which messages to verify are required to ensure that each vehicle receives appropriate awareness about neighbouring vehicles. This paper presents a novel scheme to select important safety messages for verification in vehicular ad hoc networks (VANETs). The proposed scheme uses location and direction of the sender, as well as proximity and relative-time between vehicles, to reduce the number of irrelevant messages verified (i.e., messages from vehicles that are unlikely to cause an accident). Compared with other existing schemes, the analysis results show that the proposed scheme can verify messages from nearby vehicles with lower inter-message delay and reduced packet loss and thus provides high level of awareness of the nearby vehicles.

## 1. Introduction

Vehicular ad hoc networks (VANETs) aim to improve road safety and traffic efficiency by providing communications among vehicles and between vehicles and infrastructure. In order to exchange messages about traffic conditions, an important requirement is to verify that messages are from valid sources. A general approach is for the transmitting devices to cryptographically sign and optionally encrypt messages, while the receiving devices verify the signatures and optionally decrypt the messages [1,2,3,4]. One application requiring safety messages is cooperative safety driving. The application needs to be aware of the statuses of other vehicles (e.g., position, velocity and direction) in order to warn the driver about possible safety incidents. Many proposed standards require vehicles to periodically broadcast safety or awareness messages, for example, Basic Safety Message (BSM) in Wireless Access in Vehicular Environment (WAVE) standard [3], or Cooperative Awareness Messages (CAMs) in European Telecommunications Standards Institute (ETSI) standard [4].

According to WAVE, each vehicle is recommended to broadcast safety messages to its one-hop neighbours at intervals of 100 ms or 300 ms. This could lead to a large number of safety messages to be verified. For example, on a dense highway with a broadcast interval of 100 ms, a vehicle may receive 3000 safety messages per second. Verification of a signed safety message involves time consuming cryptographic operations. With the time to verify an elliptic-curve-based digital signature around 2 ms (in 2009–2013, it was measured to take vehicles 4.97 ms on average [5,6]), the verification rate can be much lower than the rate messages are received [3,7,8,9,10,11]. To cope with this problem, the receiving vehicles could verify as many messages as possible and accept the rest without verification or simply discard them. Accepting messages without verification could cause security issues, making the system vulnerable to attacks, while discarding messages may result in loss of important information. Since a vehicle may not be able to verify all received messages, there is no guarantee that the verified messages sent to the application layer will give appropriate awareness about critical situations in time.

Road accidents mostly occur between nearby vehicles; therefore, a vehicle should have higher awareness of the situations in its immediate vicinity. For roads with no barriers between vehicles moving in opposite directions, each vehicle must pay more attention to the vehicles moving toward it in the opposite direction. The existing receiver-based message prioritization schemes proposed in literatures [7,8,9,10,11], however, have not addressed this issue. This paper presents a novel receiver-based message prioritization scheme for safety applications. The main contributions of this paper are:

- Our proposed method provides higher awareness of nearby vehicles and opposite-direction vehicles than the existing methods. This is achieved by prioritizing messages based on location and direction of the sender (represented as quadrant of the transmitter) and relative-time between vehicles. The awareness is measured via multiple metrics such as packet loss and delay to verify the messages from neighbouring vehicles.
- We also consider human reaction time (the time to react to a sudden event such as the sudden braking of the vehicle in front). To accommodate for this reaction time, the proposed method gives the highest priority to messages sent from very near vehicles, independent of their speed or acceleration, since unexpected events can happen with them. The area containing these vehicles is called danger-zone. Since vehicles travel at varying speeds, the density of nearby vehicles is also varied. Therefore, the proposed method adjusts the radius of the danger-zone according to the current average vehicle density.

This paper is organized as follows. Section 2 presents related work. Section 3 provides background assumptions on technologies and standards used in our system. The details of the proposed RTZ scheme will be presented in Section 4. Section 5 describes the experimental setup and evaluation metrics. Section 6 presents the analysis results of comparing RTZ to other existing techniques. Lastly, Section 7 concludes this paper.

## 2. Related Work

Techniques for overcoming the mismatch between message arrival rate and verification rate depend on message prioritization, which can be broadly classified, depending on where the prioritization happens, as: (1) prioritization at a transmitter and (2) prioritization at a receiver.

### 2.1. Transmitter-Based Prioritization Schemes

In the first approach, transmitting vehicles use quality of service (QoS) mechanisms such as IEEE 802.11e [12] to give more important messages higher transmission opportunity [3,13,14,15,16,17,18]. The WAVE protocol [3] provides quality of service at MAC layer by adopting the Enhanced Distributed Channel Access (ECDA) with four separate buffers (AC3, AC2, AC1 and AC0, in a descending order of priority) for prioritizing transmitting messages. Messages in the buffer with higher priority (e.g., AC3) will have more chance to access the channel. Eenennaam et al. [13] proposed the Oldest Packet Drop (OPD) buffering mechanism at the transmitter to increase the freshness of the messages sent. OPD scheme is better than the prioritization scheme in the WAVE protocol, in which messages are transmitted in first come first serve fashion and new messages are dropped when the transmit buffer is full. However, with this scheme, in very dense traffic, the freshness of messages may decrease at the receiver due to queuing and processing delay there. 

In [15], Wang et al. proposed to modify the arbitration inter-frame spacing of ECDA for WAVE to guarantee that the messages in the higher priority buffer are sent before those in other lower priority buffers. This scheme could increase delay of some relevant safety messages, which are put in low priority buffers. Schmidt et al. [14] proposed dynamically adjusting the beacon transmission rate based on traffic density, while maintaining appropriate accuracy to increase the performance of VANETs in high-density traffic condition. However, this scheme could decrease the number of safety messages and cause some applications to not receive enough safety messages to raise the alarm in critical situations. The dynamic adaptation of transmission power has been proposed in [16,17,18] to enhance the performance information dissemination in VANETs. The approaches used factors such as traffic density to adjust transmission power and some of the approaches adjusted the contention window size to give more priority to emergency messages.

### 2.2. Receiver-Based Prioritization Schemes

Although prioritization at transmitters can reduce the number of broadcast messages in the network, the prioritization does not take into account the receiver’s capacity nor the messages sent by neighbouring vehicles. Therefore, message prioritization at the receiver’s side is also required. In this technique, the receiver determines which messages are more important and relevant by using criteria such as motion, position and access category.

To enhance the security and scalability of the system, Raya et al. [19] proposed a verification scheme which chooses messages randomly from the buffer. Although this method has been used in several authentication schemes [20,21,22] due to its simplicity, a main drawback of this method is that some relevant and important messages may not be verified or verified too late. In a batch verification scheme, such as [21,23,24,25], a vehicle collects messages for some period of time and verifies all collected messages in a batch at once. An advantage of doing a batch verification is to reduce the verification time per message. However, this scheme has several drawbacks, that is, (1) a single false signature can cause unsuccessful verification of the entire batch, (2) there is additional delay for collecting messages for verification and (3) the bilinear pairing-based signature verification algorithm used in this scheme has high computational complexity [26].

Li and Chigan [9] proposed a probabilistic verification scheme in which the probability of a message being verified depends on its rank, which depends on distance and a fixed probability threshold to reduce the number of messages to be verified. In this scheme, messages from nearby vehicles and messages from vehicles that have few hops to receiver have higher probability of being verified than messages from further vehicles. However, in a high-density traffic area, there exists very low probability that messages from distant vehicles will get verified, while some of messages from nearby vehicles may still not be verified due to the probabilistic nature. In a low-density traffic scenario, Li and Chigan scheme yields similar performance as the random verification scheme [15]. 

Biswas et al. [7,8] proposed a probabilistic verification scheme where the rank of a message is based on the position, acceleration and velocity of the source vehicle. To rank a message, the message will be passed through three bloom filters [27]. Each bloom filter checks an assigned portion of safety messages against the existing entries in them. The outcome of the bloom filters is used in a binary decision tree [28] to achieve the final rank *k*. After that, messages associated to each rank will be randomly processed with a specific verification probability. The drawback of this technique is that the bloom filter has several performance limitations, as discussed in [29] and also probabilistic verification scheme may cause some close by and relevant messages not to be verified. 

In [10,11], signal strengths are used for ranking messages to be verified. Based on k-mean clustering algorithm, signal strengths are used to categorize the messages into five safety areas mapped to each level of the multi-level priority queue. A drawback of the scheme is that signal strength can vary significantly due to obstacles and environment. In addition, if the transmitter uses dynamic transmission power for transmitting messages, the approach may not be suitable. The paper reported that the area classification accuracy is around 63.2%, whereas 87.3% accuracy can be achieved when only vehicles within 50 m radius are considered.

Our proposed receiver-based method provides higher awareness of nearby vehicles and opposite-direction vehicles than the mentioned methods above. This is achieved by prioritizing messages based on location and direction of the sender (represented as quadrant of the sender) and relative-time between vehicles.

## 3. System Model

### 3.1. Case Scenario

**Figure 1:**
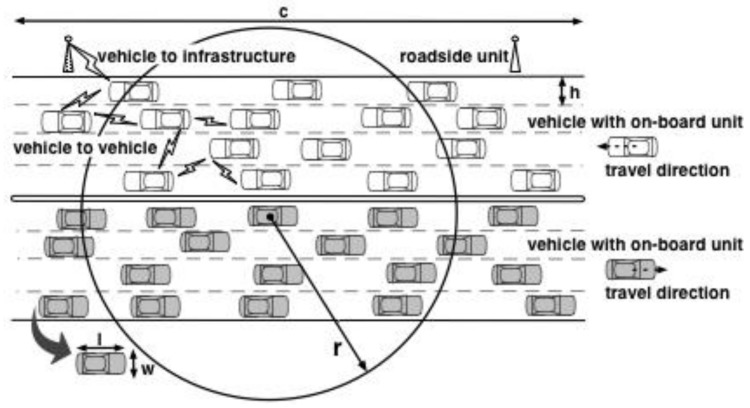
A typical road scenario with vehicular ad hoc network (VANET) system considered in this paper.

In VANETs, each vehicle is generally equipped with an on-board unit, which has limited processing and storage capabilities. There are fixed roadside units along the road, with which vehicles may communicate directly or indirectly via other vehicles. Figure 1 illustrates the road scenario considered in this paper. We assume a straight dual carriageway, which may not have any barrier separating the vehicles traveling in opposite directions: the grey vehicles are moving in the right direction, while the white ones in the left direction. The length of the road is *c* km with *e* lanes (width *h* per lane) for each direction. The importance of quadrants, defined in Section 4.1, can be adjusted to cope with a single carriageway.

Each vehicle has a wireless transceiver that can communicate with other vehicles or roadside units up to a range of *r* m. The vehicle can receive all messages sent by all vehicles within its communication range *r*. Each vehicle has length *l* and width *w*. Vehicle speed may vary from the minimum speed (*s_min_*) to the maximum speed (*s_max_*). The current velocity and acceleration of vehicle *i* are denoted as *v_i_* m/s and *a_i_* m/s^2^, respectively. Vehicles may change lanes while traveling along the road. All vehicles are equipped with sensors to determine their location (longitude *x*, latitude *y*), heading, velocity and other necessary physical movement parameters. 

### 3.2. Wireless Communications

**Figure 2:**
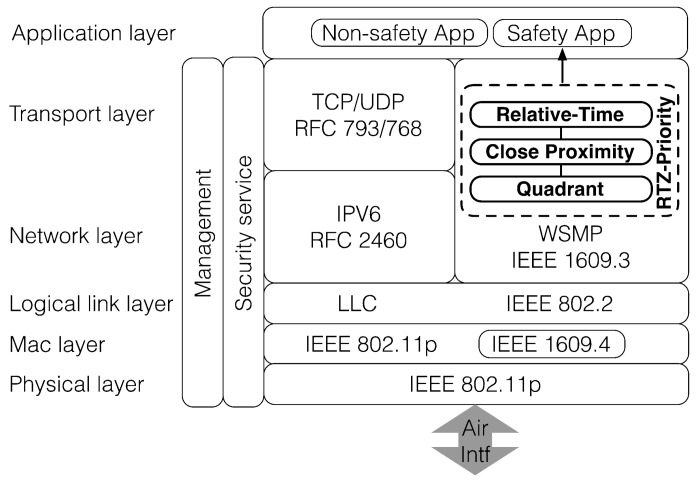
Relative time and zone (RTZ) prioritization scheme in the wireless access in vehicular environment (WAVE) protocol stack.

We assume that the architectural components (i.e., on-board unit, roadside unit and wireless interface) of a VANET is compatible with the IEEE 1609 family of standards for WAVE [3]. Figure 2 shows a layered view of WAVE. IEEE 1609 defines IEEE 802.11p [30] as the wireless communications technology and supports delivery of safety messages using the WAVE Short Message Protocol (WSMP). A common message set used by safety applications is defined by SAE J2735 [31]. WAVE uses IEEE 802.11p [30] at the physical layer for enabling wireless access and IEEE 802.11e [12] at the MAC layer to provide quality of service.

In general, an on-board unit uses a single First-In-First-Out (FIFO) buffer, at the network and transport layer, for handling receiving messages from entities in a VANET. In addition to TCP/IP protocol stack, WSMP safety messages are included as a part of a new stack defined by IEEE 1609.3. This paper proposes the Relative Time and Zone (RTZ) prioritization scheme (highlighted in bold) to extend how the receiver processes the received WSMP messages.

### 3.3. Safety Applications

SAE J2735 over Dedicated Short Range Communication (DSRC) [32] is a standard for messaging in VANETs. This standard defines fifteen types of messages for use in VANET communications. Basic Safety Message (BSM) is an important message type used by cooperative safety driving applications or vehicle-to-vehicle safety applications (for the rest of this paper, a BSM is referred to as safety message or message). The default size of each safety message is 254 bytes.

Vehicles typically broadcast safety messages at an interval of either 100 ms or 300 ms to inform neighbours about their statuses. To avoid delay, there is no acknowledgment or handshaking in safety message delivery. They are broadcast to all wireless (one-hop) neighbours. Based on the WAVE standard, vehicles can communicate up to the range of 1000 m. A vehicle may use the maximum communication range for specific purposes such as sending emergency messages or routing messages. The National Highway Traffic Safety Administration (NHTSA) suggested in the DOT-HS-812014 [33] the operational range of up to 300 m for vehicle-to-vehicle communication. Each safety message contains information about the status of a vehicle, such as position, direction, velocity, acceleration and optional information such as event flags. Periodic broadcasting of safety messages by all vehicles allows other vehicles to be aware of nearby vehicles. Safety applications that use safety messages include forward collision warning, blind spot warning, lane change warning and pre-crash warning [34,35].

## 4. Relative-Time and Zone Prioritization

The proposed RTZ scheme prioritizes safety messages based on three metrics, namely, location and direction of the sender (represented as quadrant of the sender), close proximity (danger zone) and relative-time between vehicles. 

### 4.1. Ranking Based on Direction and Location

We use the direction (same or opposite) and location (in front of or behind) of a transmitting vehicle relative to the receiving vehicle to group messages into four quadrants:

*Quadrant 1*: If the transmitting vehicle is traveling in the same direction and is in front of the receiving vehicle, then there is a possibility of the receiving vehicle approaching the transmitting vehicle. Messages from such senders are important for the receiver to be aware of overtaking or potential accidents with vehicles ahead.

*Quadrant 2*: If the transmitting vehicle is traveling in the same direction and is behind the receiving vehicle, then there is a possibility of the transmitting vehicle approaching the receiving vehicle. Messages from the transmitting vehicle are important for the receiver to be aware of overtaking or potential accidents with vehicles behind it. 

*Quadrant 3*: If the transmitting vehicle is traveling in the opposite direction and is in front of the receiving vehicle, then there is a possibility of the receiving and transmitting vehicles approaching each other. Depending in the road configuration (median strip, barrier between directions), messages from the transmitting vehicle may be important for the receiver to be aware of the approaching sender. 

*Quadrant 4*: If the transmitting vehicle is traveling in the opposite direction and is behind the receiving vehicle, then there is very low chance that those two vehicles will interact. Messages from the transmitting vehicle are less likely to be of value to the receiver compared to the above three cases.

**Figure 3:**
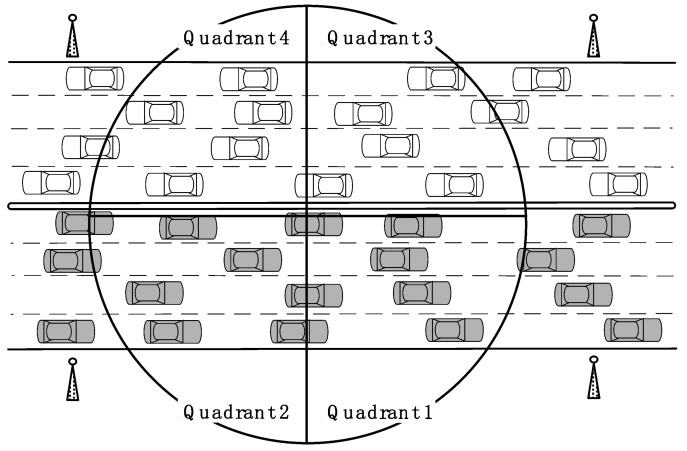
Quadrant concept, where quadrant 4 has the lowest priority in the RTZ scheme, while the other quadrants have the same priority.

Although this classification does not fully capture the importance of safety messages, it can be combined with close proximity and relative-time rankings. An example of the four quadrants is given in Figure 3. As messages are delivered across a single hop only, the quadrants are bounded by the range of the receiving vehicle.

As safety messages contain the transmitting vehicles’ positions and directions, the receiving vehicle can determine the quadrant for a received message by comparing these values with its own position and heading angle (determined by positioning sensors in the on-board unit).

### 4.2. Ranking Based on Close Proximity

Nearby vehicles are more likely to cause safety incidents or have urgent information compared to distant vehicles. Therefore, distance between vehicles has been used as a metric for safety messages prioritization [9,10]. In this study, distances between vehicles are further classified into adaptive discrete zones, adjusted by the density distribution of vehicles in the network. We refer to this ranking metric as close proximity. For ranking safety messages based on close proximity, the receiving vehicle divides the area of communication (1-hop) into *n* zones. The safety messages from closer zones to the receiving vehicle will have higher priority for message verification.

**Figure 4:**
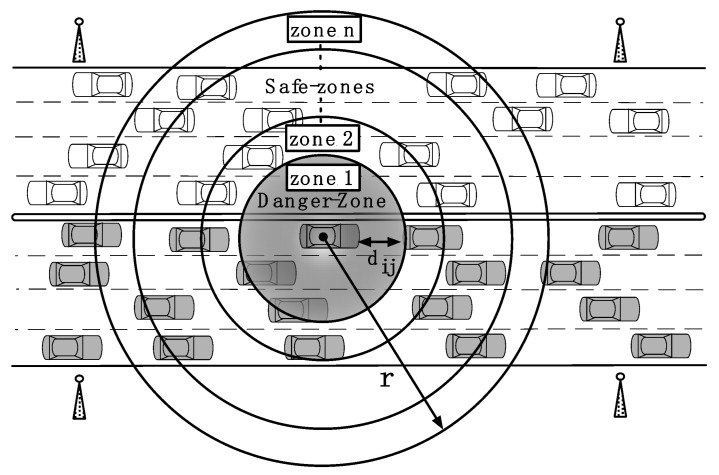
Close proximity concept. The first area is the danger-zone which has the highest priority for message verification. Other areas are safe-zones where messages are verified based on resource availability.

As shown in Figure 4, the first zone is called the *danger-zone*. We define the danger-zone of a vehicle *i* such that vehicle *i* can completely verify all messages from the vehicles in the danger-zone within a pre-specified maximum delay time *δ*. Messages from vehicles in the danger-zone are all considered important, regardless of the current direction and relative position (in front, behind) of the transmitting vehicle. Since nearby vehicles can suddenly change their velocity and acceleration, they are given the highest priority. Other zones will be referred to as *safe-zones.*

A key design parameter is the size of the danger-zone. In this study, we assume *δ* is significantly smaller than the shortest reaction time and the message generation interval *τ*. According to [36,37], human reaction time may vary between 0.4 and 2.7 s. Hence, to allow the driver to start reacting as soon as possible, the pre-specified delay time *δ* should be small compared to the interaction time. According to the WAVE standard, each vehicle generates one BSM message every either *τ* = 100 or 300 ms. This message generation interval is the same for all vehicles in the system. Hence, we also need to make sure that the receiving vehicle can process the messages from vehicles in the danger-zone in time. That is, we need δ<τ.

To guarantee that all messages from the danger-zone are verified within *δ* on average, the number of vehicles in the danger zone, $N_{dz}$, can be defined as:

(1) $$N_{dz}=\frac{\delta }{p}$$

where p is the processing time to verify a message. There can be at most δ/p vehicles because each vehicle generates only one message within the time *δ* and each message requires time p to verify.

The radius of the danger-zone, $r_{dz}$, can be determined from the average vehicle density. This can be determined from the number of vehicles in the transmission range *r*, $N_{net}$, defined as:

(2) $$N_{net}=\frac{\lambda_{rx}}{\lambda_{tx}}=\tau \;\lambda_{rx}$$

where $\lambda_{rx}$ is the rate of messages received (can be calculated by each receiving vehicle). These received messages are generated by $N_{net}$ transmitting vehicles at a transmission rate $\lambda_{tx}=1/\tau$. 

Based on the uniform distribution assumption (in the area $\pi r^{2}$), the radius of the danger zone is defined as:

(3) $$r_{dz}=r\sqrt{\frac{N_{dz}}{N_{net}}}=r\sqrt{\frac{\delta }{p\;\tau \lambda_{rx}}}$$

Note that the higher the vehicle density, the smaller the radius of the danger-zone.

Assume the radius of other zones are incremented with the same scale, the maximum number of zones, M, is

(4) $$M=\lceil \frac{r}{r_{dz}}\rceil$$

where ⌈.⌉ is the ceiling operator.

If a transmitting vehicle *i* is at distance $d_{ij}$ from the receiving vehicle *j*, the transmitting vehicle *i* is in zone *z* of the receiving vehicle *j* where

(5) $$z=\{\begin{array}{ll} \lceil \frac{M\;d_{ij}}{r}\rceil , & d_{ij}\leq r\\ M, & d_{ij}>r \end{array}$$

where $d_{ij}$ is the distance between the two vehicles, z=1 for the *danger-zone* and 2 to M for the *safe-zones*.

### 4.3. Ranking Based on Relative-Time

In addition to distance, the rate of change in vehicle positions should also be considered in the ranking metrics since it may affect the future distance between vehicles. We combine distance, velocity and acceleration to determine the relative-time between a sender and a receiver. 

If vehicle *i* has a smaller relative-time than vehicle *j* with respect to a receiving vehicle, *rx*, then vehicle *i* will reach *rx* first (and potentially cause a safety incident) if both vehicles continue with the same motion. Therefore, messages from vehicles with a smaller relative-time to the receiving vehicle are considered more important and should be verified first.

To calculate relative-time, we assume acceleration of both vehicles will remain constant and that all vehicles in the same direction are in the same lane (i.e., vehicles are projected onto a single straight line). The next position of a vehicle *i* is predicted as:

(6) $${x^{\prime }}_{i}=x_{i}+v_{i}\left(t^{\prime }-t\right)+\frac{1}{2}a_{i}{\left(t^{\prime }-t\right)}^{2}$$

where $x_{i}$ is the initial position, $x_{i}^{\prime }$ is the final position, t is the initial time, $t^{\prime }$ is time elapsed, $a_{i}$ is the initial acceleration and $v_{i}$ is the initial velocity. Since the receiving vehicle is an observer of the situations, the value for t is 0, that is:

(7) $${x^{\prime }}_{i}=x_{i}+v_{i}t\prime +\frac{1}{2}a_{i}{t^{\prime }}^{2}$$

The relative-time between two vehicles, $t^{\prime }$, is the time it takes for those two vehicles to reach the same position (i.e., $x_{rx}^{\prime }=x_{tx}^{\prime }$). Given the current position, velocity and acceleration of a transmitting vehicle *tx* and a receiving vehicle *rx*, $t^{\prime }$ can be found by solving:

(8) $$\frac{1}{2}\left(a_{rx}-a_{tx}\right){t^{\prime }}^{2}+\left(v_{rx}-v_{tx}\right)t\prime +x_{rx}-x_{tx}=0$$

The drawbacks of using only relative-time for message ranking are twofold. First, due to buffering and processing delay, the assumption that acceleration is constant may not be valid over a long period of time. Second, close vehicles with similar motion (i.e., same direction, same velocity and acceleration), although the messages between them are very important, may have high relative-time.

### 4.4. Combined Final Ranking

To enhance the message ranking algorithm, we combine the three formerly proposed ranking metrics for final message ranking. The messages will be inserted into the receiver buffer according to this rank. The highest rank is at the head of the buffer. When a receiving vehicle receives a safety message (*SM*), it extracts the necessary information, such as position, acceleration and velocity, of the transmitting vehicle. This information, plus its own status, are used to determine the zone (z), quadrant (q) and relative-time (t′) of the transmitting vehicle. 

The RTZ algorithm can be summarized as follows: For a receiving vehicle *i*,

1. If a safety message *SM* comes from the vehicles in the safe zone of *i* and belongs to quadrant 4, then *SM* has the lowest priority and will be inserted at the end of the buffer.
2. Otherwise, *SM* will be inserted into the buffer based on the zone *z* (from closest to distant zone).
3. If there are multiple messages in the buffer with the same zone, the messages will be arranged based on the relative time t′ (from low to high value).
4. To ensure that only recent messages are verified, messages that are older than a specified time (i.e., 2 s) will be dropped.

In RTZ, the receiver gives safety messages coming from close vehicles higher priority than those from distant vehicles (where “close” and “distant” are zones). Within each zone, safety messages are arranged based on their relative-time from lowest to highest. Finally, safety messages from quadrant 4 are set to lower priority than all other quadrants.

## 5. Experimental Setup and Evaluation Metrics

To illustrate the effectiveness of RTZ against other methods, a network simulation was conducted using NS3.19 [38] with VANET-Highway version 2 module [39]. A high-density highway scenario is considered with parameter values listed in Table 1. 

The distance-log propagation path loss model, which is the default channel model in NS3, was used to simulate the wireless channel on the highway. The path loss (dB) is defined as:

(9) $$L=L_{0}+10n\log_{10}\left(\frac{d}{d_{0}}\right)$$

where n is the path loss exponent, $d_{0}$ is the reference distance (m), $L_{0}$ is the path loss (dB) at the reference distance and *d* is the distance (m). To reflect the non-line-of-sight and obstructions from other vehicles, the default value n = 3 was used (note that for line-of-sight free space path loss, *n* = 2).

The communication range in VANETs is not easy to determine. This is because VANETs uses 5.9 GHz spectrum for communication and hence the factors such as buildings, plants, obstacles and interference with other radio frequency cause effect on the range of communication in VANETs. In previous studies [9,33,40,41,42], the range of 300 m was used as the one-hop communication range of vehicle. This range is consistent with the experimental measurements of DSRC performance in [43,44,45].

The VANET-Highway module in NS3 allows vehicles to adjust their speeds and lanes during simulation, with the aim of maintaining a minimum distance between two vehicles to be greater than a value (30 m in our simulation). To avoid the vehicles at the start/end of the 2500-m highway (which have fewer neighbours than those in the middle) from biasing the results, data were collected only from the vehicles within the middle 1500 m.

Since vehicles were injected at the start of the highway in each direction, to ensure the highway is full of vehicles, the first 120 s of simulation data from 300 s simulation was discarded. We ran 20 independent simulation runs. To save computational time, in each run, we collected data from 100 randomly selected vehicles as representatives of all vehicles. Each performance metric described later is an average of these 20-run results. To show variation in the results, we calculated the 95% confidence intervals for all the results.

We compared RTZ with other receiver-based prioritization schemes [3,9,19] that prioritize messages according to different criteria, namely,

1. First In First Out (FIFO): a baseline approach giving no priority to received safety messages in a buffer, as used in [3].
2. Last In First Out (LIFO): new messages are verified first. Although not used by other schemes, we include it for comparison. LIFO is a reasonable scheme because the freshest message is verified first.
3. Serve In Random Order (SIRO): messages received are selected at random to be verified, as proposed in [19]. The purpose of this algorithm is to allow high reliability and scalability.
4. Li and Chigan [9] ’s method (for convenience, referred to as Li scheme in this paper): a probabilistic scheme, where messages are ranked based on the distance and number of vehicles between the receiving and transmitting vehicles. The probability of a message being verified depends on the rank and a fixed parameter *p*. That is, messages from vehicles, which are close by and have less neighbours are assigned a higher rank and have a higher probability of verification.

The first three schemes are basic scheduling algorithms (where the prioritization criteria do not depend on the information about the transmitting vehicles), while the last scheme is a priority queuing scheme (where the receiver ranks messages based on the information from the transmitting vehicle). To be realistic, the messages with age more than two seconds are dropped from the buffer. We assume that all the periodically transmitted safety messages have the standard size of 254 bytes and the communication overhead of all the schemes is the same.

To show the effectiveness of the different prioritization schemes, the following five performance metrics are used:

1. *Packets Verified*: the number of packets that are verified in each scheme. The verified portion of packets is different for each scheme according to their design. As a result, the verified messages have impact on delay and awareness of vehicles.
2. *Delay*: the time from the generation of a message to the time that the message is verified. The message delay should be low so that the safety applications could provide a proper response to safety incidents.
3. *Packet Loss*: the number of packets that are dropped due to the scheme design, buffer overflow, or their age exceeds the specified lifetime. This metric also has an effect on awareness of vehicles in the vicinity.
4. *Inter-Message Delay* [46,47]: the delay between two consecutive messages that come from the same transmitting vehicle and are verified at the receiving vehicle. This metric measures whether the verified messages are up-to-date.
5. *Cooperative Awareness Quality Level (AQL)* [48]: the average awareness of *V* randomly selected vehicles in zone z at the *T* specified time instants, defined as
  (10) $$AQL\left(T,z\right)=\frac{\sum_{j=1}^{T}\sum_{i\in V}A_{z}^{t\left(j\right)}\left(i\right)}{T\times V}$$
  where $A_{z}^{t\left(j\right)}\left(i\right)$ is the awareness by the receiving vehicle *i* of the vehicles in zone z at time *t*(*j*). The awareness $A_{z}^{t\left(j\right)}\left(i\right)$ is the ratio of the vehicles in zone *z* that are aware by *i* (i.e., the messages of these vehicles are verified by (*i*) at time *t*(*j*) and the total number of vehicles in zone *z* at that time instant. A set of *V* receiving vehicles are randomly selected and the records of their awareness are tracked for *T* different time instants, *t*(1) to *t*(*T*). AQL(T,z) is an average of these individual awareness records over all vehicles and time instants.

## 6. Simulation Results

### 6.1. Safety Message Receiving Rate in a High-Density Traffic Scenario

**Figure 5:**
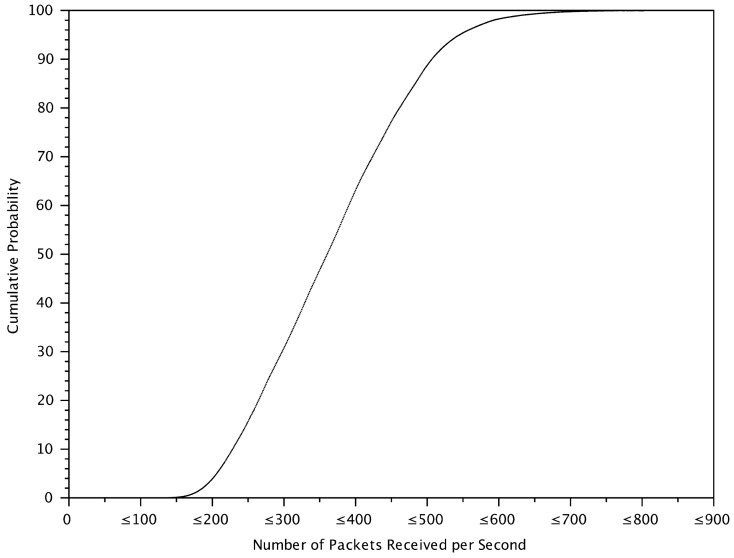
The cumulative probability distribution of the number of packets received per second by each vehicle.

Figure 5 shows the cumulative probability distribution of the safety message receiving rate in the simulation. With a signature verification time of an average of 5 ms [5,6] or equivalently 200 messages per second, about 95% of the time the verification rate is too low to verify all messages. This figure confirms that message prioritization is required.

Note that although the 5 ms verification time is the number in 2009–2013, today’s number might be 2 ms, which gives 500 messages verified per second. This means 15% of the time vehicles receive messages more than verification capability. Furthermore, in a general situation, when the message verification gets faster, each vehicle tends to generate messages at a faster rate. Therefore, vehicles should carefully select relevant messages for verification.

### 6.2. A Comparison of the Percentage of Verified Messages with Li Scheme

**Figure 6:**
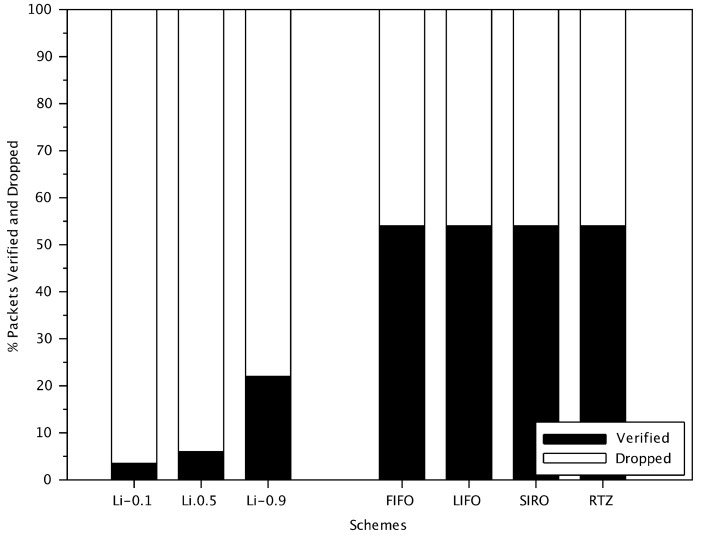
Comparison of percentage of verified messages of Li scheme with different filtering probabilities (i.e., 0.1, 0.5 and 0.9) with other schemes in a dense traffic area.

Figure 6 shows the percentage of verified messages and dropped messages by the receivers for the different schemes (messages not verified are dropped). FIFO, LIFO, SIRO and RTZ verify the same number of messages. For these schemes, the number of messages verified depends only on the processing time at the receiver. However, for Li scheme, the percentage of messages verified also relies on the filtering probability and the message rank.

In Figure 6, we show the performance of Li scheme with filtering probabilities (i.e., 0.1, 0.5 and 0.9). Messages from vehicles that are close by and have less intervening vehicles have a higher probability of verification. In a dense network if the distance between transmitting and receiving vehicles increases, the number of vehicles between them will also increases, thus the message is less likely to be verified. For example, in Li scheme with probability 0.1 (shown as Li-0.1 in Figure 6), a message with rank 5 is verified with a probability of $0.1^{5}$.

The results show that with a filtering probability less than 1, Li scheme verifies significantly fewer messages than other approaches (just 22% of the received messages with a probability of 0.9). With a filtering probability of 1, Li scheme is equivalent to FIFO and, although not shown in Figure 6, verifies the same number of messages as others. In the subsequent results, unless otherwise noted, we focus on Li scheme with a probability of 0.9 as this value still makes Li scheme different from FIFO while still verifies many messages.

### 6.3. Effectiveness of Danger-Zone for Different Quadrants in RTZ

**Figure 7:**
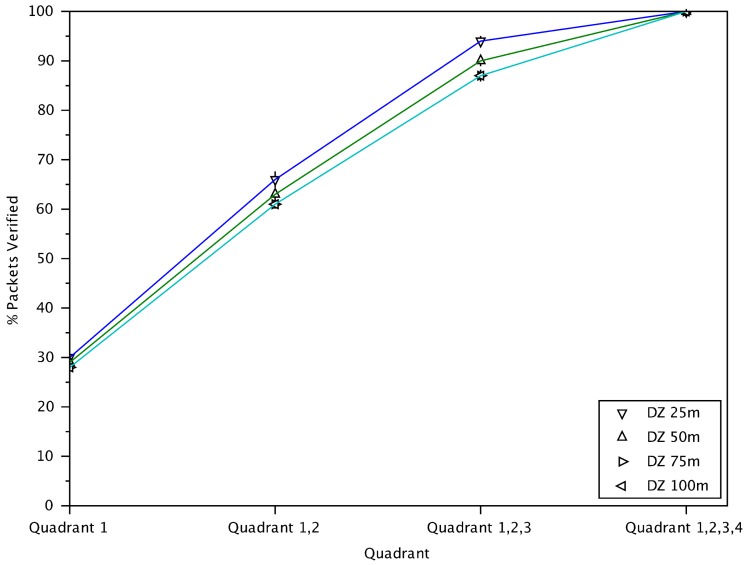
The cumulative percentage of verified messages from different quadrants for RTZ with different danger-zone radii.

A key design feature of RTZ is the danger-zone in which messages from transmitting vehicles in the zone, regardless of the quadrants, are treated with the highest priority. Also, messages from quadrant 4 are less important compared to others. Figure 7 illustrates the cumulative percentage of verified packets from different quadrants using RTZ with varying sizes of the danger-zones (i.e., 25 m to 100 m). The results show that approximately the same percentage of messages from quadrants 1, 2 and 3 are verified and less for quadrant 4. In our proposed scheme, quadrant 4 contains vehicles which travel in the opposite direction and are behind the receiving vehicle, where there is very low chance of interaction. Messages from these vehicles are less likely to be of value to the receiver. With a smaller danger-zone radius (i.e., 25 m), fewer messages from quadrant 4 are verified. In the subsequent analysis, we will use a danger-zone radius of 25 m, as it effectively filters out messages from the less important vehicles in quadrant 4 in a high-density traffic condition.

### 6.4. Verification Rate versus Quadrant

**Figure 8:**
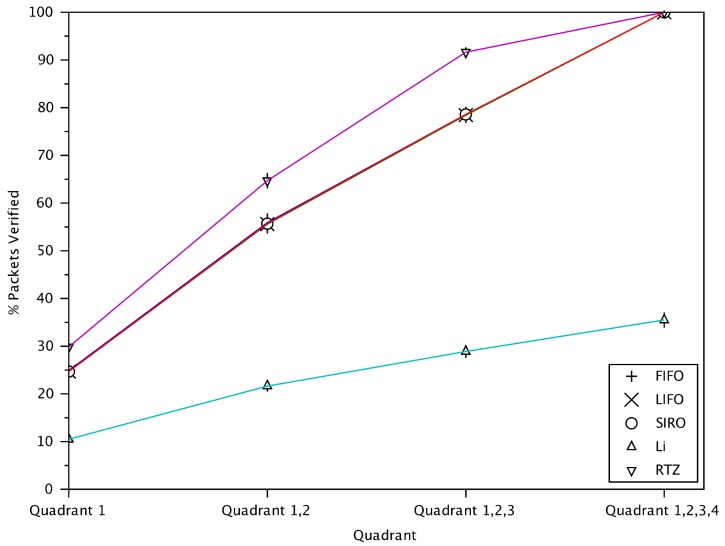
The cumulative percentage of verified messages from different quadrants.

Figure 8 shows the cumulative percentage of verified messages from different quadrants for different schemes. FIFO, LIFO and SIRO results are almost identical. Each of the 4 quadrants hold approximately 25% of the transmitting vehicles. Although Li scheme verifies less messages in total (about 32%) compared to other schemes, the verified messages are still distributed approximately equally across the 4 quadrants. This is because those approaches do not consider the location and direction of transmitting vehicles relative to the receiving vehicle. 

For RTZ, most of the verified messages are from quadrants 1, 2 and 3, while only about 5% of the verified messages are from quadrant 4. This demonstrates a key feature of RTZ, that is, the ability to focus verification on the important quadrants, 1, 2 and 3. Quadrant 4, with vehicles behind and in the opposite direction, is considered less important. Therefore, fewer messages from vehicles in that quadrant are verified with RTZ. Note that as RTZ also gives higher priority to the danger-zone around a receiving vehicle, some messages from quadrant 4 in the danger-zone are still verified.

### 6.5. Verification Rate versus Distance between Vehicles

**Figure 9:**
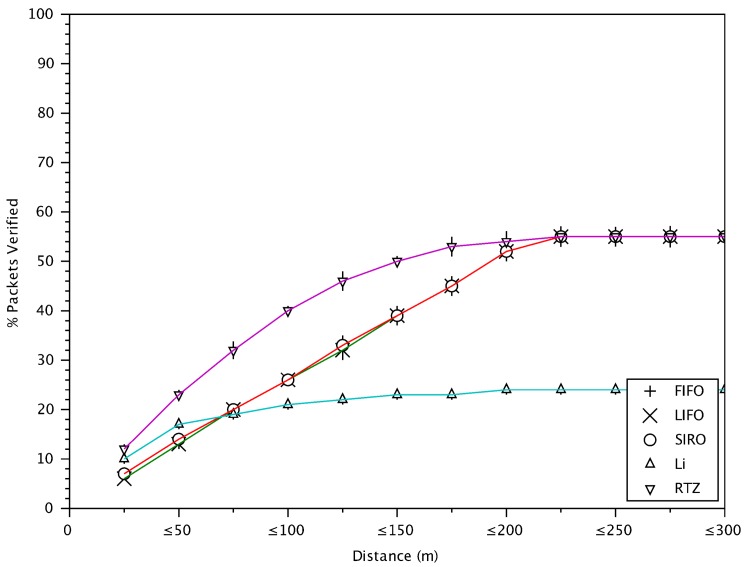
The cumulative percentage of verified messages with respect to distances between transmitting and receiving vehicles.

Figure 9 shows the percentage of verified messages (compared to the total number of messages received) versus the different distances between transmitting and receiving vehicles. RTZ achieves the best performance in verifying messages from nearby vehicles as it gives higher priority to closer-by vehicles. On the other hand, FIFO, LIFO and SIRO give almost identical performance and the same verification rate for vehicles at all distances since they do not consider the vehicle information. In RTZ, the danger-zone is the first zone (25 m) around a receiving vehicle. A receiving vehicle verifies all messages originating from this zone. RTZ verifies 8% and 2% more messages in the danger-zone compared to basic approaches and Li scheme. In the Li scheme, most of the safety messages verified from the vehicles in range of 100 m from the receiving vehicle. Since Li scheme ranks messages based on number of neighbours between sender and receiver and distance, it may not be suitable for high-density areas since messages from vehicles further away are verified with very low probability. In addition, the randomness in selecting messages means sometimes messages from close vehicles will not be verified. Considering the results in Figure 8 and Figure 9, RTZ is promising as it verifies messages from nearby vehicles and less of those from quadrant 4

### 6.6. Verification Rate versus Delay of Verified Messages

**Figure 10:**
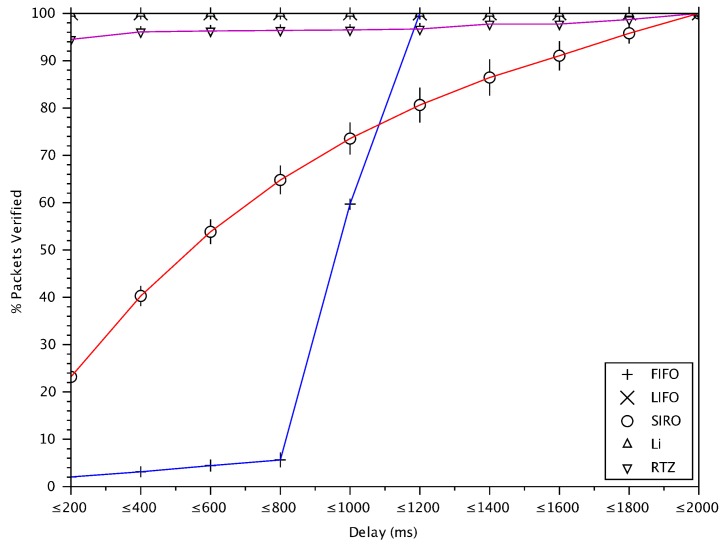
The cumulative percentage of verified messages versus delay of verified messages.

As VANETs are highly dynamic, it is important to verify messages with a small delay, otherwise the information contained may be out-of-date. Recall that we have a 2 second lifetime for messages (after which they are dropped), however preferably messages are verified in a much shorter time. Figure 10 shows the cumulative percentage of verified messages with respect to different delay values for different message verification schemes. FIFO is the worst scheme since it gives priority to the oldest messages. LIFO and Li scheme can verify almost 100% of messages with a delay less than 200 ms. In LIFO, new messages are verified first resulting in a very low delay. Li scheme has a high drop rate and the receiver buffer is empty most of the time, therefore most of the messages are verified with low delay. Our proposed RTZ verifies approximately 95% of the messages with less than 200 ms delay.

### 6.7. Delay of Verified Messages versus Distance between Vehicles

**Figure 11:**
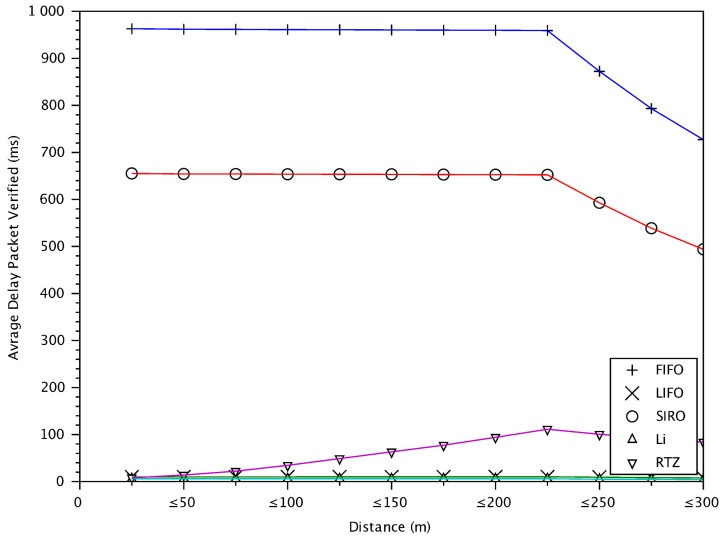
Average delay of verified messages versus distances between transmitting and receiving vehicles.

Since safety applications require message delay less than 100 ms [35], Figure 11 shows that RTZ meets this requirement for almost all vehicles in the communication distance. We can see again that LIFO and Li give the best message delay while FIFO the worst, for the same reasons discussed for the previous figure.

### 6.8. Inter-Message Delay and Packet Loss versus Distance between Vehicles

**Figure 12:**
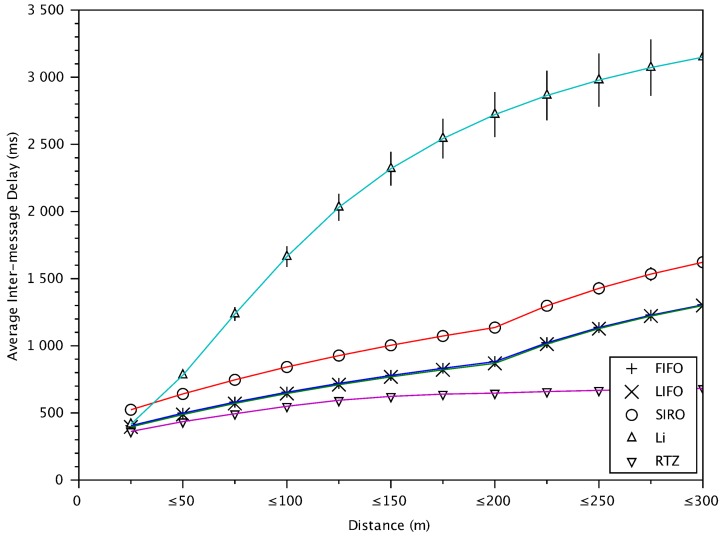
The inter-message delay for verified messages versus distances between transmitting and receiving vehicles.

Since safety applications also require that the verified information comes at a regular and short interval, Figure 12 shows that RTZ gives the lowest inter-message delay (among all verified messages) for all vehicles within the communication range. Since messages are generated at every 300 ms at a transmitting vehicle, the inter-message delay must be at least 300 ms. Since RTZ verifies almost all messages originated within the danger-zone (<25 m), the inter-message delay is about 300 ms as shown in the figure. For further vehicles, some of their messages are not verified in time and hence the inter-message delay is more than 300 ms. The further the vehicles, the more the unverified messages and hence the higher the inter-message delay. As expected, Li scheme gives the worst performance since it drops a lot of messages from the far away vehicles.

**Figure 13:**
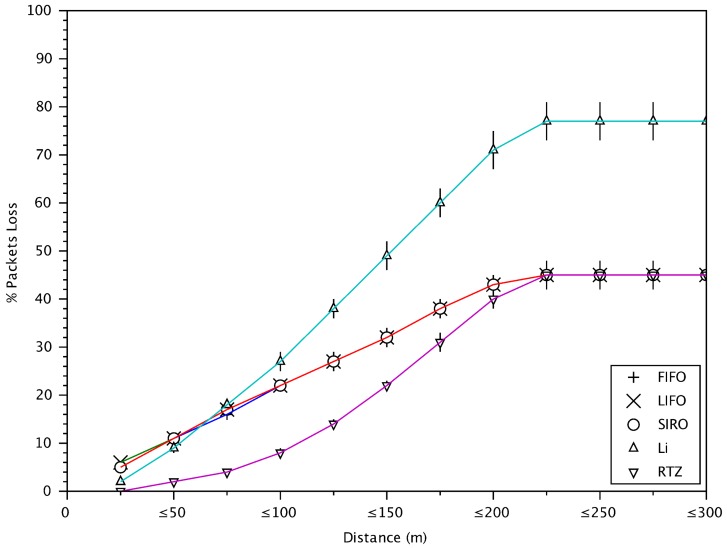
The cumulative percentage of packet loss with respect to distances between vehicles.

Figure 13 shows the packet loss versus distance. Now we can see the reason why the inter-message delay of RTZ in Figure 12 is about 600 ms for the vehicles at distance greater than 200 m. The reason is shown in Figure 13, where the packet loss for messages from those vehicles is about 50%. That means, on average one of two messages are not verified and hence the inter-message delay is twice the message generation time. For the vehicles at closer distance, the packet loss is smaller and hence the inter-message delay is smaller. 

### 6.9. Awareness Quality Level versus Distance between Vehicles

**Figure 14:**
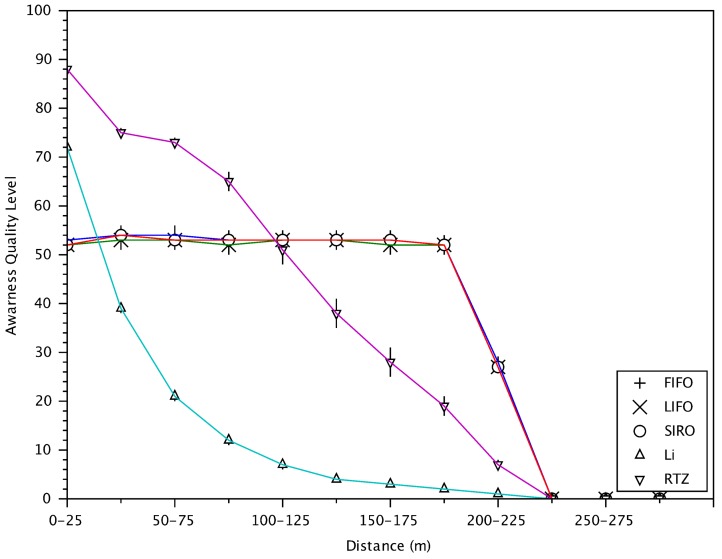
Awareness quality level (AQL) of receiving vehicle with respect to distances between vehicles.

Figure 14 shows the awareness index, AQL, defined in (10) versus distance. As expected, RTZ allows vehicles to be more aware of the nearby vehicles. It achieves the highest AQL for vehicles within 100 m. Since FIFO, LIFO and SIRO do not care about vehicle location, their awareness scores are the same and the same for all distances up to 200 m. Interestingly but not surprisingly, Li gives the minimum awareness at any distance as it verifies only some messages. 

### 6.10. An Analysis of Close Distance Results

This section gives a summary of the results for different schemes for a close distance of less than 25 m where accidents are most likely to occur. The verification rate of FIFO message verification scheme, which is the baseline and default mechanism of WAVE [3] protocol, is 8% less than that of RTZ. In FIFO, the average delay of verified messages with respect to distance of transmitting vehicles is on average 960 ms which is significantly higher than RTZ. FIFO has poor awareness for vehicles close by (i.e., 38% less than RTZ). As a result, FIFO scheme may not provide appropriate awareness for cooperative safety driving applications. SIRO verifies 8% fewer messages from close by vehicles compared to RTZ with 660 ms higher delay on average. The average inter-message delay for SIRO is also 200 ms higher than RTZ. It also has poorer awareness of vehicles in the vicinity by ~38% compared to RTZ. The verification rate of Li scheme (using a filter probability of 0.9) is 32% less than RTZ. In Li scheme, packet loss rate and inter-message delay are increasing rapidly with increasing distance between receiving and transmitting vehicles due to the probabilistic nature of the scheme. The high packet loss and probabilistic verification cause Li scheme to achieve 14% poorer awareness for nearby vehicles compared to RTZ. For messages from vehicles in a close distance, our proposed RTZ message prioritization scheme achieves better performance compared to the baseline FIFO scheme and the state-of-the-art approaches in terms of the message verification rate, packet loss, inter-message delay and awareness.

### 6.11. Experiments with Moderate and Low Density Networks

So far, we have presented the simulation results for highways with a high density of vehicles (the minimum distance between vehicles is 30 m). We chose to study in detail such highly dense highways since the message arrival rate is much greater than the verification rate and hence a good prioritization scheme is needed and its performance is clearly pronounced. We have also performed simulation study for highways with a moderate density (about 45 m minimum distance between vehicles) and low-density (about 60 m minimum distance). The results for the moderate density network for RTZ and other schemes show similar results (in term of trends and relative performances) as those for the highly dense highways. This is because the message arrival rate still exceeds the verification rate, although the excess time happens less frequently and the excess is smaller as the density decreases. As the density decreases, the necessity of a good prioritization scheme decreases since more and more messages can be verified. Therefore, the results for the low-density network among the different schemes show similar trend and performance.

## 7. Conclusions

In high density VANETs, the rate at which safety messages are received can exceed the digital signature verification rate for typical on-board units. We proposed the Relative-Time and Zone (RTZ) message prioritization scheme that determines which arriving safety messages should be verified first, based on the quadrant of the transmitting vehicles, close proximity and the relative-time between vehicles. RTZ filters incoming safety messages based on the quadrant of the transmitting vehicle, treating messages that arrive from vehicles that are traveling in the opposite direction and behind the receiving vehicle as the least important. Then, messages are grouped into zones based on distances between transmitting and receiving vehicle and the network density. Finally, we consider each vehicle’s position, velocity and acceleration to determine the relative-time between vehicles. The messages from vehicles with a smaller relative-time are considered more important in each zone. 

To illustrate the effectiveness of the proposed RTZ scheme, we use simulation to compare RTZ against the default FIFO and other schemes. The simulation results show that RTZ achieves the best performance. In terms of awareness RTZ achieves the highest average awareness (AQL = 88%) for nearby vehicles (e.g., 25 m). This means that 88% of the nearby vehicles, within the danger-zone, are being aware. In addition, RTZ gives the highest message verification rate for nearby to distant vehicles with negligible packet loss for vehicles in the danger-zone and a little more packet loss for nearby vehicles. For the verification delay, RTZ achieves an average delay less than 40 ms for vehicles within 100 m distance and 100 ms delay for distant vehicles, while the inter-message delay is less than 700 ms across all distances. 

In summary, our proposed RTZ can provide high awareness and high rate of messages verified from nearby vehicles with short delay. It is a good candidate for message prioritization scheme for cooperative driving safety applications. However, since RTZ relies on not-yet-verified information of the vehicles (such as position and speed), it might be vulnerable to attacks from somebody who knows the prioritization scheme. Further work is to counter such an attack.

## Figures and Tables

**Table 1:** Simulation setup parameters.

Parameter	Value
Number of lanes	4 lanes/direction
Lane width	3 m
Road length	2500 m
Space between vehicles	30 m
Number of vehicles entering highway	4 vehicles/s
Vehicle velocity	65–85 km/h
BSM interval, τ	300 ms
BSM lifetime	2 s
BSM processing time, *p*	5 ms
BSM size	254 bytes
Buffer capacity	200 pkts
Danger-zone range, *r_dz_*	25 m
Data rate	6 Mb/s
Radius of the largest zone, *r*	300 m
Maximum delay time *δ* for messages from vehicles in danger-zone	0.1 s
